# Survivin-specific T-cell reactivity correlates with tumor response and patient survival: a phase-II peptide vaccination trial in metastatic melanoma

**DOI:** 10.1007/s00262-012-1266-9

**Published:** 2012-05-08

**Authors:** Jürgen C. Becker, Mads H. Andersen, Valeska Hofmeister-Müller, Marion Wobser, Lidia Frey, Christiane Sandig, Steffen Walter, Harpreet Singh-Jasuja, Eckhart Kämpgen, Andreas Opitz, Marc Zapatka, Eva-B. Bröcker, Per thor Straten, David Schrama, Selma Ugurel

**Affiliations:** 1 Department of Dermatology, Medical University Graz, Auenbruggerplatz 8, 8010 Graz, Austria; 2 Center for Cancer Immune Therapy, Department of Hematology, Herlev University Hospital, Herlev, Denmark; 3 Department of Dermatology, Julius-Maximilians-University, Würzburg, Germany; 4 Immatics Biotechnologies GmbH, Tübingen, Germany; 5 Department of Dermatology, University Hospital Erlangen, Erlangen, Germany; 6 Institute of Clinical Transfusion Medicine and Hemotherapy, Julius-Maximilians-University, Würzburg, Germany; 7 Division of Theoretical Bioinformatics, German Cancer Research Center, Heidelberg, Germany

**Keywords:** Melanoma, Survivin, T-cell reactivity, Therapy, Peptide vaccination

## Abstract

**Background:**

Therapeutic vaccination directed to induce an anti-tumoral T-cell response is a field of extensive investigation in the treatment of melanoma. However, many vaccination trials in melanoma failed to demonstrate a correlation between the vaccine-specific immune response and therapy outcome. This has been mainly attributed to immune escape by antigen loss, rendering us in the need of new vaccination targets.

**Patients and methods:**

This phase-II trial investigated a peptide vaccination against survivin, an oncogenic inhibitor-of-apoptosis protein crucial for the survival of tumor cells, in HLA-A1/-A2/-B35-positive patients with treatment-refractory stage-IV metastatic melanoma. The study endpoints were survivin-specific T-cell reactivity (SSTR), safety, response, and survival (OS).

**Results:**

Sixty-one patients (ITT) received vaccination therapy using three different regimens. 55 patients (PP) were evaluable for response and survival, and 41/55 for SSTR. Patients achieving progression arrest (CR + PR + SD) more often showed SSTRs than patients with disease progression (*p* = 0.0008). Patients presenting SSTRs revealed a prolonged OS (median 19.6 vs. 8.6 months; *p* = 0.0077); multivariate analysis demonstrated SSTR as an independent predictor of survival (*p* = 0.013). The induction of SSTRs was associated with gender (female vs. male; *p* = 0.014) and disease stage (M1a/b vs. M1c; *p* = 0.010), but not with patient age, HLA type, performance status, or vaccination regimen.

**Conclusion:**

Survivin-specific T-cell reactivities strongly correlate with tumor response and patient survival, indicating that vaccination with survivin-derived peptides is a promising treatment strategy in melanoma.

**Electronic supplementary material:**

The online version of this article (doi:10.1007/s00262-012-1266-9) contains supplementary material, which is available to authorized users.

## Introduction

Treatment for metastatic melanoma currently undergoes a transformation, changing the rigid scheme of dacarbazine as standard treatment in all stage-IV melanoma patients, attributed with a very low response rate and an extremely poor survival, into new, individualized therapeutic strategies. For the first time since decades, new drug therapies succeeded in demonstrating a significant survival benefit [1–3] in contrast to the numerous clinical trials reported before [4]. On the one hand, kinase inhibitors like the anti-BRaf V600E agent vemurafenib clearly showed an improved survival in patients carrying the respective gene mutation [2]. On the other hand, the immunomodulating antibody ipilimumab, an enhancer of T-cell-mediated immune responses, also demonstrated a prolongation of survival in metastatic patients [3]. The latter agent is of particular interest, because it is supposed to generate persistent anti-tumoral immune responses and to hereby elicit long-term disease control and prolonged survival in the corresponding patients. Following these promising findings, T-cell-based treatment strategies, which mainly are active tumor-specific vaccinations, got again into the focus of clinical testing and evaluation in melanoma. The ultimate goal of these efforts would be to develop a therapeutic strategy consisting of a vaccination generating an efficient T-cell response, which will thereafter be enhanced or at least maintained by non-specific immune modulation.

With regard to an active, antigen-specific immunotherapy, the identification of defined melanoma-associated antigens opened the opportunity to develop anti-melanoma vaccines [5]. In this respect, immunization with HLA-restricted peptide epitopes derived from differentiation antigens is a strategy that has been vigorously pursued. Initial clinical trials using gp100 peptide vaccination plus IL-2 in stage-IV melanoma achieved objective responses in 12/32 patients (42 %) [6]. Unfortunately, many of the thereafter studied vaccines aiming to induce immune responses against differentiation antigens failed to demonstrate clinical efficacy. Reviewing 440 patients, only four complete and nine partial responses were observed, rendering an objective response rate of 3 % [7].

In the present study, we vaccinated melanoma patients not against a differentiation antigen, but against the oncogenic molecule survivin. Survivin is a bifunctional inhibitor-of-apoptosis protein that plays a key role in the protection of tumor cells from apoptosis. Accordingly, a potential down-regulation of survivin expression as a strategy of immune escape would severely impair a tumor cell’s survival capacity. Moreover, survivin is overexpressed in melanoma, as well as in most cancer entities of epithelial and hematopoietic origin, and its overexpression is associated with disease progression and poor prognosis in the respective patients [8–10], which makes survivin an excellent candidate for therapeutic vaccinations against cancer [11, 12]. Preclinical studies using a survivin-specific DNA vaccine showed vaccine-induced immune responses eradicating pulmonary metastases in lung cancer patients [13]. Encouraged by these findings, we developed a peptide-based vaccine against survivin [14] and found this vaccine to induce T-cell responses in heavily pretreated melanoma and pancreatic cancer patients without significant toxicity [15, 16]. Furthermore, in situ peptide/HLA-A2 multimer staining revealed infiltrating survivin-reactive CD8+ T cells in soft tissue metastases of vaccinated patients. Driven by these promising results, the present phase-II study was intended to investigate the correlation between a vaccine-specific immune response and the corresponding treatment outcome. To improve the induction of survivin-specific immune responses, we twice amended the vaccination regimen. The first amendment (Regimen II) increased the frequency of vaccinations within the first 8 weeks, and the second amendment (Regimen III) introduced an upfront application of low-dose cyclophosphamide intended to deplete regulatory T cells.

## Patients and methods

### Study design

The primary endpoint of this single-arm, single-institution, prospective phase-II trial (NCT00108875; ClinicalTrials.gov) was a vaccine-specific immune response measured as ex vivo survivin-specific T-cell reactivity (SSTR). Secondary endpoints were safety, best overall response, overall survival (OS), and progression-free survival (PFS). The study endpoints were evaluated on intention-to-treat (ITT) and per-protocol (PP) basis. Patient recruitment was outlined as a total of 50 patients evaluable for response and survival. This sample size was calculated as sufficient for an exploratory analysis to draw correlations between vaccine-specific immune response and treatment outcome. The results of this analysis were intended to be implemented into the design of a currently planned randomized phase-III trial.

### Patient population

Patients with histologically confirmed metastatic melanoma were enrolled in accordance with the following main eligibility criteria: stage-IV disease following AJCC criteria [17]; at least one prior systemic therapy in stage-IV resulting in disease progression; at least one measurable target lesion according to RECIST [18]; stop of any previous anti-tumor or immunosuppressive treatment at least 4 weeks before the first vaccination; HLA type of A1 and/or A2 and/or B35; overall performance status (OPS) according to ECOG criteria ≤2; no active infection or autoimmune disease; and adequate bone marrow, hepatic, and renal functions. All types of metastatic sites were considered eligible including metastases to the brain, as well as all localizations of primary including cutaneous, mucosal, uveal, and unknown primaries. Prognostic factors of metastatic melanoma, serum lactate dehydrogenase (LDH), as well as OPS, were recorded at treatment onset. The study protocol was approved by the Institutional Review Board, and written informed consent was signed by all patients prior to enrollment.

### Vaccination therapy

Patients received vaccinations with HLA-restricted peptide epitopes derived from survivin [14]. The peptide sequences were modified in order to enhance their HLA binding affinity [19, 20]. The peptides used were FTELTLGEF (HLA-A1; PolyPeptide Laboratories, Wolfenbüttel, Germany), LMLGEFLKL (HLA-A2; Clinalfa, Sissach, Switzerland), and EPDLAQCFY (HLA-B35; PolyPeptide Laboratories), all of pharmaceutical (GMP) quality. Each vaccination comprised 100 μg of each peptide matching the patient’s HLA type emulsified in 1 ml Montanide^®^ ISA-51 (Seppic, Paris, France) and was administered by deep subcutaneous injections. Three different vaccination regimens were used in consecutive order: vaccinations in weeks 1, 2, and 5, followed by 4-week intervals (Regimen I); weekly vaccinations in week 1–8, followed by 4-week intervals (Regimen II); and the schedule of Regimen II preceded by a single i.v. dose of cyclophosphamide 250 mg/m^2^ 24 h prior to the first vaccination (Regimen III). Toxicity was evaluated using common toxicity criteria (CTC) 2.0 (http://ctep.cancer.gov/reporting/ctc.html).

### Ex vivo detection of survivin-specific T-cell reactivity

Enzyme-linked immunospot (ELISPOT) assays were used to quantify IFNγ-releasing survivin-specific effector T cells in samples of peripheral blood mononuclear cells (PBMCs) as described previously [21]. Briefly, nitrocellulose-bottomed 96-well plates (MultiScreen MAIP N45, Millipore, Schwalbach, Germany) were coated with an anti-IFNγ antibody (1-D1K, Mabtech, Stockholm, Sweden), and non-specific binding was blocked using AIM-V (Life Technologies, Gaithersburg, MD). Lymphocytes were isolated from heparinized peripheral blood samples of study patients and subsequently incubated overnight at 37 °C at different cell concentrations together with the respective HLA-matched survivin epitope-specific peptides and T2 cells. The peptides used in the assay were the same as those used for patient vaccination. After two washing procedures, the biotinylated detection antibody (7-B6-1-Biotin, Mabtech) was added. Specific binding was visualized using alkaline phosphatase–avidin together with the respective substrate (Life Technologies). The reaction was stopped on the appearance of dark purple spots as a measure of IFNγ-release, which was quantified using the AlphaImager System (Alpha Innotech, San Leandro, CA). Reactivity was considered positive, if the IFNγ release of cells incubated with a specific peptide was more than tripling the release of the same cells incubated without a peptide in at least two independent experiments.

### MHC multimer assay

Peptides for HLA-class-I multimers were ILKEPVHGV from HIV-1-RT-476-484, LTLGEFLKL from human parental survivin 96-104, and its modified form LMLGEFLKL. Biotinylated recombinant peptide–HLA-A*0201-monomers and multimers were produced as previously described [22]. Dual MHC multimer assessment was performed 12 days after a single round of in vitro sensitization as described previously [23]. Briefly, PBMCs were pulsed with 10 μg/ml readout class-I peptides for 2 h, then pelleted, resuspended, and cultured for 13 days in X-vivo 15 (Lonza, Verviers, Belgium) plus 10 % heat-inactivated human AB serum (C.C.Pro, Neustadt, Germany), 2 mM l-glutamine (Lonza), and 40 U/ml IL-2 (Novartis, Munich, Germany). Harvested PBMCs were stained first with Live/Dead Aqua (Invitrogen, Karlsruhe, Germany), multimer-PE, and multimer-APC (each at 5 μg/ml MHC), followed by anti-CD8-FITC and anti-CD3-PacificBlue (Becton–Dickinson, Heidelberg, Germany). Cells were fixed and analyzed on a LSRII cytometer (Becton–Dickinson), gated on live CD8+ CD3+ lymphocytes.

### Assessment of tumor response and survival

Patients who completed at least 28 days of vaccination, corresponding to two vaccinations in Regimen I and four vaccinations in Regimens II and III, respectively, were considered evaluable for treatment response and survival (PP). Tumor response was assessed by CT and/or MRI imaging in 8-week intervals and evaluated according to RECIST [18]. Complete (CR) and partial (PR) responses were combined as objective response (OR). Patients who died from melanoma rapidly after treatment onset were considered as progressive disease (PD). Best overall response was defined as the best response recorded between the start and the end of treatment; best overall responses of stable disease (SD) or better were considered as progression arrest (CR + PR + SD) [18]. All CT and MRI scans from patients showing progression arrest were retrospectively reviewed by an independent radiologist. OS and PFS were measured from the date of first vaccination until the date of death or disease progression, respectively. If no such event occurred, the date of the last patient contact was used as endpoint of survival assessment (censored observation).

### Statistical analysis

Fisher’s exact test was used to compare T-cell reactivities, tumor response rates, and toxicities between groups. Survival curves and median survival times were calculated using the Kaplan–Meier method for censored failure time data. The logrank test was used for comparison of survival probabilities between groups. 95 % confidence intervals for median survival were calculated using the method of Brookmeyer [24]. Multivariate testing using the proportional hazards model of Cox was applied to test for independent predictors of survival in adjustment with the clinical covariates age, gender, and disease stage (M category). All *p* values are two-tailed and unadjusted for potential multiple comparisons to allow a hypothesis-building exploratory data analysis; *p* < 0.05 was considered statistically significant.

## Results

### Patient characteristics and study flow

**Fig. 1:**
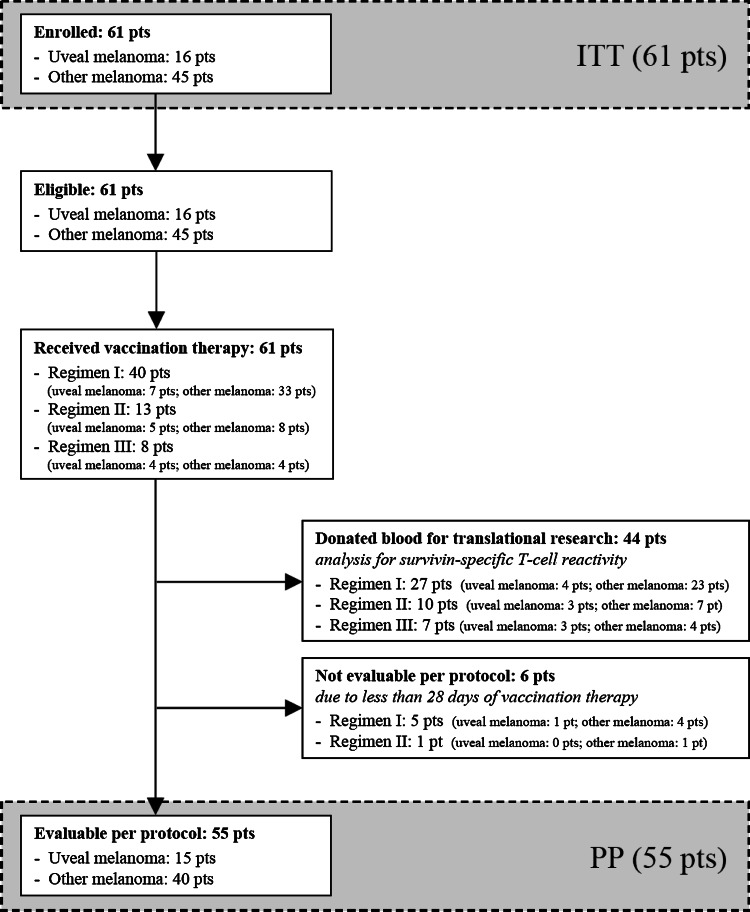
Schematic presentation of the study flow (CONSORT diagram). *ITT* intention-to-treat, *PP* per-protocol

Between 03/2003 and 11/2007, 61 patients were enrolled into the study (ITT); detailed patient characteristics are presented in Tables 1 and S1 (S, supplementary; all supplementary materials available online)*;* the distribution on the different vaccination regimens can be seen in Fig. 1 and Table S1. All 61 patients met the eligibility criteria and started vaccination therapy within 1 week following enrollment. 6/61 patients (9.8 %) had to be excluded from PP analysis due to less than 28 days on treatment (Fig. 1); 55/61 patients (90.2 %) were evaluable for treatment response and survival (PP).

**Table 1:** Patient characteristics at enrollment, treatment efficacy, and outcome

	ITT 61 (100.0 %)	PP 55 (100.0 %)
Gender		
Male	39 (63.9 %)	35 (63.6 %)
Female	22 (36.1 %)	20 (36.4 %)
Median age/years (range)	62.5 (28.4–82.7)	61.3 (28.4–82.7)
HLA type^a^		
A1	20 (32.8 %)	19 (34.5 %)
A2	42 (68.9 %)	32 (58.2 %)
B35	15 (24.6 %)	15 (27.3 %)
Serum LDH		
≤UNL	38 (62.3 %)	37 (67.3 %)
>UNL	23 (37.7 %)	18 (32.7 %)
Performance status (ECOG)		
0	45 (73.8 %)	44 (80.0 %)
1	12 (19.7 %)	10 (18.2 %)
2	4 (6.5 %)	1 (1.8 %)
M category (AJCC)		
M1a	6 (9.8 %)	6 (10.9 %)
M1b	9 (14.8 %)	9 (16.4 %)
M1c	46 (75.4 %)	40 (72.7 %)
Inflammatory reaction at vaccination sites		
Yes	18 (29.5 %)	18 (32.7 %)
No	43 (70.5 %)	37 (67.3 %)
Survivin-specific T-cell reactivity (SSTR)^b^		
Positive	13 (21.3 %)	13 (23.6 %)
Negative	31 (50.8 %)	28 (50.9 %)
Not assessed	17 (27.9 %)	14 (25.5 %)
Best overall response^c^		
CR	1 (1.6 %)	1 (1.8 %)
PR	3 (4.9 %)	3 (5.5 %)
SD	7 (11.5 %)	7 (12.7 %)
PD	50 (82.0 %)	44 (80.0 %)
Objective response (CR + PR)	4 (6.6 %)	4 (7.3 %)
Progression arrest (CR + PR + SD)	11 (18.0 %)	11 (20.0 %)
Median progression-free survival months (95 % CI)^d^	2.8 (2.2–3.9)	3.0 (2.4–4.1)
Median overall survival months (95 % CI)^d^	9.1 (6.1–11.3)	9.8 (6.4–11.9)

*ITT* intention-to-treat, *PP* per-protocol, *LDH* lactate dehydrogenase, *UNL* upper normal limit, *ECOG* Eastern Cooperative Oncology Group, *AJCC* American Joint Committee on Cancer, *CR* complete response, *PR* partial response, *SD* stable disease, *PD* progressive disease, *CI* confidence interval

^a^Multiple entries possible

^b^Survivin-specific T-cell reactivities (SSTR) were quantified by ELISPOT as described in "Patients and methods" and classified as positive or negative as described in “Results”

^c^Best overall response was defined as the best tumor response recorded from the start of treatment until removal of the patient from the trial

^d^Survival was measured from the date of first vaccination until the date of death or disease progression, respectively

### Survivin-specific T-cell reactivity (SSTR)

**Fig. 2:**
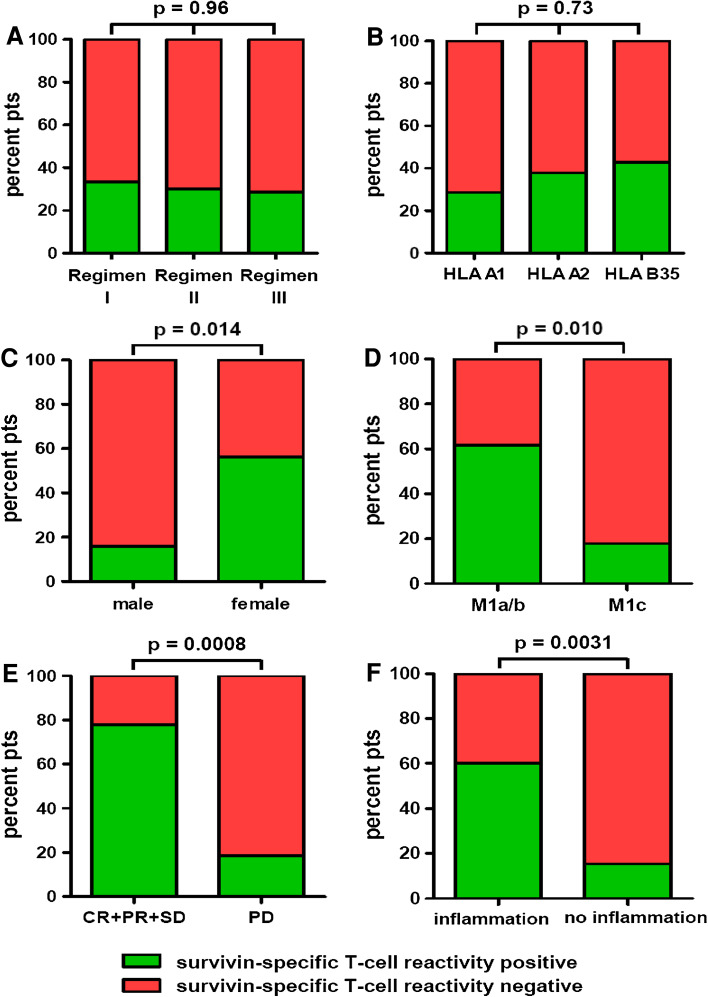
Survivin-specific T-cell reactivities (SSTR) of the per-protocol population (55 patients) as detected by ELISPOT, diagramed by **a** vaccination regimens; **b** patients’ HLA type; **c** patients’ gender; **d** M category according to AJCC criteria; **e** best overall response grouped as progression arrest (CR + PR + SD) and progression (PD); and **f** inflammatory reaction at the vaccination sites. Patients demonstrating a positive detection of SSTR at at least one time point during the first 16 weeks of ongoing vaccination were defined positive (*green bars*); patients without this reactivity were considered negative (*red bars*). Fisher’s exact test was used to compare T-cell reactivities between groups; *p* values are provided above the corresponding *bars*

41/55 PP patients (74.5 %; Regimen I = 24 pts; Regimen II = 10 pts; Regimen III = 7 pts) consented in peripheral blood withdrawal and analysis of PBMCs by ex vivo ELISPOT for SSTRs before the first vaccination (at baseline) and every 8 weeks thereafter until termination of study treatment. Patients demonstrating a positive ex vivo detection of SSTRs at at least one time point during the first 16 weeks of ongoing vaccination (either at baseline and/or at week 8 and 16, respectively) were defined “positive”; patients without positive reactivity were considered “negative.” 13/41 patients (31.7 %) presented positive SSTRs during vaccination. These reactivities in the majority of patients were first detected at 8 weeks following the first vaccination and stayed positive for up to 60+ months; two of the 13 patients (15.4 %) showing positive SSTRs were already positive at baseline and stayed positive during ongoing vaccination. The presence of SSTRs was neither influenced by the vaccination regimen (*p* = 0.96; Fig. 2a) nor by the patients’ HLA type (*p* = 0.73; Fig. 2b). Interestingly, female patients presented SSTRs significantly more often than males (*p* = 0.014; Fig. 2c). Patients in stages M1a/b more often revealed SSTRs than patients in stage M1c (*p* = 0.010; Fig. 2d); moreover, a trend toward less frequent SSTRs was observed in patients with elevated serum LDH compared to patients with normal LDH levels (*p* = 0.16; data not shown). Patients with uveal melanoma also showed a trend toward less frequent SSTRs compared to patients with melanomas of other origins (*p* = 0.056; data not shown). Patients’ OPS (*p* = 0.57) and age at therapy onset (*p* = 0.41) had no significant impact on SSTRs (data not shown).

### MHC multimer staining

**Fig. 3:**
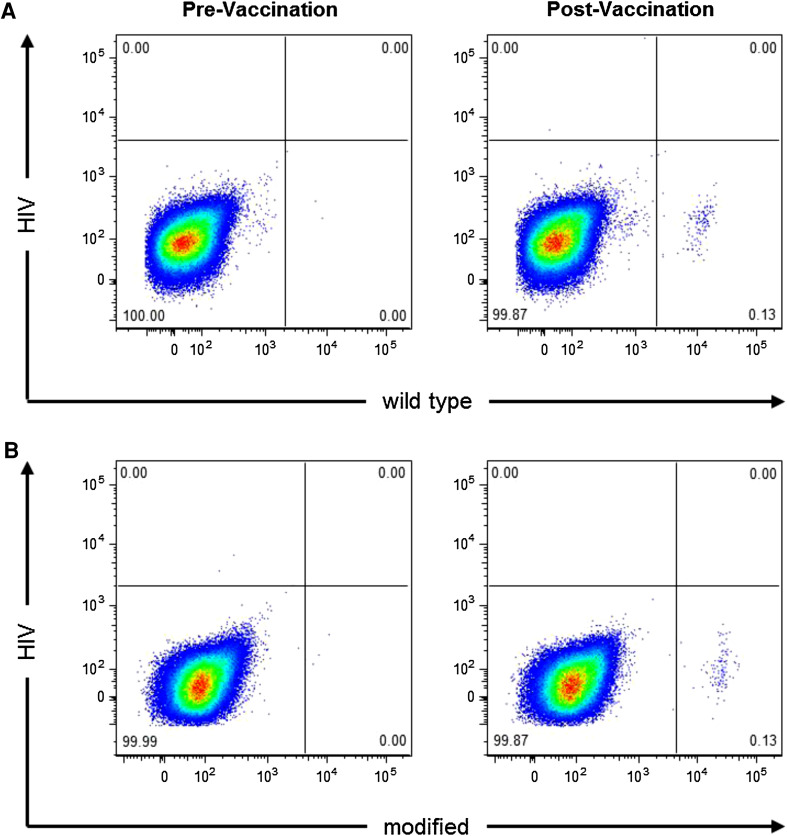
Vaccination-induced CD8+ T cells recognize the modified and wild-type HLA-A2-restricted survivin epitopes. PBMCs drawn from a HLA-A2+ patient before (*left panels*) and after 8 weeks (*right panels*) of vaccination in Regimen I were incubated with the modified survivin peptide LMLGEFLKL, the wild-type survivin peptide LTLGEFLKL, and the HIV-derived peptide ILKEPVHGV as negative control. Cells were stained with the HLA multimers HIV (A*0201-ILKEPVHGV) and wild-type survivin (A*0201-LTLGEFLKL) (**a**) or with HLA multimers HIV and modified survivin (A*0201-LMLGEFLKL) (**b**). Cells were analyzed by flow cytometry and gated on live CD8+ CD3+ lymphocytes

Flow cytometry analysis using soluble survivin peptide–MHC multimers, which specifically interact with respective T-cell receptors, were performed in exemplary patients who showed positive SSTRs at 2 months after onset of vaccination. Comparison of the results obtained from the use of HLA multimers, which braced the modified or the wild-type survivin epitopes, respectively, revealed that T cells reactive against either multimer could be detected among the PBMCs of vaccinated patients (Fig. 3).

### Tumor response and patient survival

The database was frozen in December 2008 with a median follow-up time of 45 months. Tumor response to treatment is presented in Table 1; no significant differences could be observed between the three treatment regimens. The characteristics of patients showing a progression arrest are given in Table 2. Considering the PP population, 49 deaths occurred, and six patients were still alive with four of them receiving ongoing vaccination. A detailed presentation of OS and PFS is provided in Table 1. With regard to known prognostic factors of metastatic melanoma, we observed a favorable OS in patients with normal versus elevated serum LDH (*p* = 0.0009; Figure S1A), in patients at stage M1a/b versus M1c (*p* = 0.0074; Figure S1B), and in patients presenting an OPS = 0 versus OPS > 0 (*p* = 0.21; Figure S1C). Neither vaccination regimen nor patients’ gender or HLA type had a significant impact on overall survival (data not shown). Patients with uveal melanoma revealed an impaired survival compared to patients with melanoma originating from other localizations (*p* = 0.039; Figure S1D).

**Table 2:** Characteristics of patients with progression arrest

Sex	Age (years)	Localization of primary melanoma	Stage (AJCC)	HLA type	Vaccination regimen	OPS (ECOG)	Previous therapy in stage-IV	Sites of metastases	Number of vaccinations (ELISPOT)^a^	Survivin-specific T-cell reactivity vaccination sites	Inflammatory reaction at response^b^	Best overall	PFS (months)	OS (months)
m	61	Cutaneous	M1a	A1, A2	Regimen I	0	Temozolomide, interferon-alpha	LN, intramuscular	62+	Negative	Absent	PR	64+	64+
m	46	Cutaneous	M1b	A2	Regimen I	0	Temozolomide, interferon-alpha	Lung, LN	60+	Positive	Present	CR	63+	63+
f	86	Cutaneous	M1c	A2, B35	Regimen I	0	Temozolomide, interferon-alpha	Liver, LN	55+	Positive	Present	PR	58+	58+
f	52	Cutaneous	M1b	A2	Regimen I	0	Dacarbazine, radiation	Lung, LN, SC	39	Positive	Present	PR	45	48+
m	63	Uveal	M1c	B35	Regimen I	0	Imatinib	Liver, lung	11	n.a.	Absent	SD	9	31
f	73	Cutaneous	M1a	A1, B35	Regimen I	1	Dacarbazine	LN, SC	10	Positive	Absent	SD	5	28
f	78	Cutaneous	M1b	A2	Regimen I	1	Dacarbazine	Lung, LN	18	Positive	Present	SD	20	25
f	70	Cutaneous	M1a	A2, B35	Regimen II	0	Dacarbazine	LN, SC, C	18	Positive	Present	SD	8	20
m	69	Cutaneous	M1c	A2	Regimen I	0	Dacarbazine, fotemustine	Brain, lung, LN, SC	12	n.a.	Absent	SD	6	16
m	34	Cutaneous	M1b	A2, B35	Regimen III	0	Temozolomide	Lung, LN, SC	14	Positive	Absent	SD	8	15
m	62	Uveal	M1c	A2	Regimen I	0	Imatinib	Liver	8	Negative	Absent	SD	6	9

Patients are sorted by overall survival (OS, last column). Age, stage of disease, overall performance status (OPS), and sites of metastases refer to the time point of first vaccination

*AJCC* American Joint Committee on Cancer, *ECOG* Eastern Cooperative Oncology Group, *PFS* progression-free survival, *n.a.* not assessed, *LN* lymph node, *SC* subcutaneous, *C* cutaneous

^a^Patients demonstrating a positive detection of survivin-specific T-cell reactivity at at least one time point during ongoing vaccination were defined positive; patients without any positive reactivity throughout the vaccination period were considered as negative

^b^Best overall response was defined as the best tumor response recorded from the start of treatment until removal of the patient from the trial. All responses presented here were reviewed and confirmed by an independent radiologist

### Survivin-specific T-cell reactivity correlates with tumor response and patient survival

**Fig. 4:**
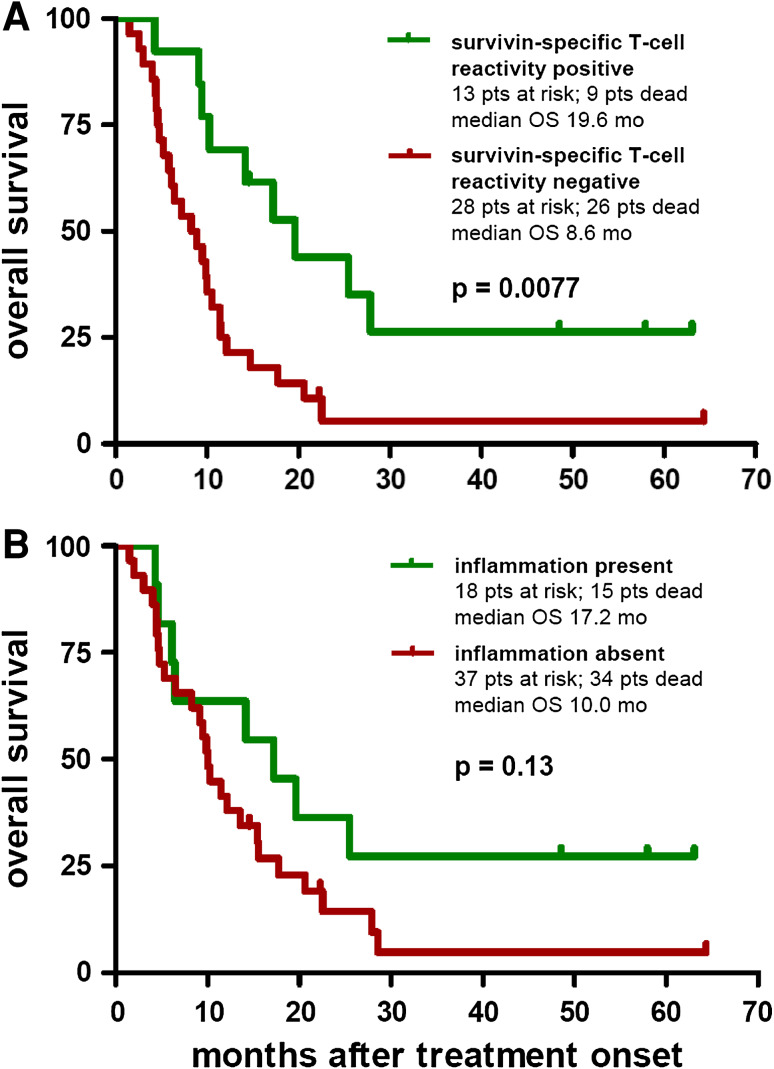
Kaplan–Meier plots depicting the probability of overall survival (OS) of the per-protocol population (55 patients) by **a** survivin-specific T-cell reactivity (SSTR) detected by ELISPOT as described in “Patients and methods” and classified as positive or negative as described in “Results”; and **b** inflammatory reaction at the vaccination sites. Differences between groups were calculated using the logrank test; *p* values are provided within the corresponding plots. Censored observations are indicated by *vertical bars*

Concerning tumor response, patients experiencing a progression arrest (CR + PR + SD) under vaccination revealed significantly more often SSTRs than patients with a disease progression (*p* = 0.0008; Fig. 2e). Moreover, patients presenting a SSTR during vaccination revealed a significantly prolonged OS compared to patients showing no survivin-specific reactivity (median 19.6 vs. 8.6 months, *p* = 0.0077; Fig. 4a). Multivariate analysis using the proportional hazards model of Cox including the parameters age, gender, disease stage (M category), and SSTR revealed SSTR (*p* = 0.013) and disease stage (*p* = 0.027) as independent prognostic predictors. Age (*p* = 0.38) and gender (*p* = 0.12) resulted as no independently significant prognostic parameters.

### Treatment-related toxicity

**Fig. 5:**
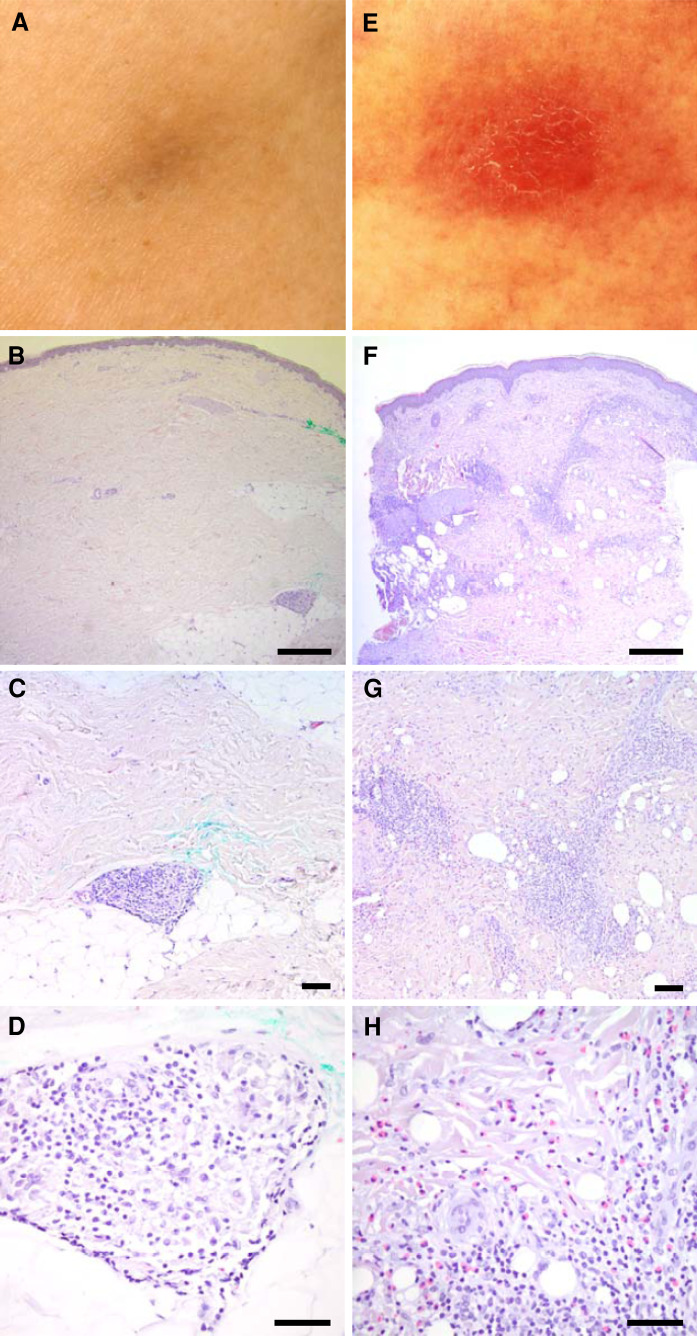
Vaccination sites in two representative patients showing a weak (**a**–**d**) and a strong (**e**–**h**) inflammatory reaction, respectively. Staining with hematoxylin and eosin. Magnification ×10 (**b**, **f**), ×20 (**c**, **g**), and ×40 (**d**, **h**)

The majority of treatment-related side effects were mild to moderate (CTC grade 1–2). The most common toxicities were fever and chills on the day of vaccination and inflammatory reactions at the injection sites characterized by erythematous, dense, and painful nodules arising in about 30 % of patients (Table 1; Fig. 5). Interestingly, the occurrence of these post-vaccination inflammatory reactions was strongly associated with the presence of SSTRs (*p* = 0.0031; Fig. 2f). Moreover, patients presenting vaccination-induced inflammatory reactions showed a trend toward a favorable survival (*p* = 0.13; Fig. 4b). CTC grade 3–4 toxicities potentially related to study therapy are summarized in Table S2. Most of these toxicities were unspecific conditions, which must be considered rather tumor-related than therapy-related. None of the observed toxicities required any action, and no significant differences could be observed between the three vaccination regimens tested (data not shown).

## Discussion

Encouraged by our first promising observation of a successful survivin peptide vaccination in heavily pretreated stage-IV melanoma patients [15], we tested its safety, immunogenicity, and clinical efficacy in the present phase-II trial. Hereby, the major goal was to show a correlation between survivin-specific immune response and treatment outcome.

Sixty-one patients (ITT) were included into this trial; 55 (PP) were evaluable for treatment response and survival, and 41/55 were evaluable for SSTR. Notably, for all patients, disease progression under the previous treatment line was confirmed by imaging studies. With >70 % of the PP population in stage M1c, >50 % harboring two or more metastatic sites, >35 % already received two or more therapies, and 15 % presenting brain metastases, the patient cohort was characterized by an extremely poor prognosis. Nevertheless, four patients (7 %) achieved an OR, and seven patients (13 %) a SD; thus, 20 % revealed a progression arrest translating into a median OS of 31.4 months. The established prognostic factors of advanced melanoma, M category, OPS, and localization of the primary, showed a significant impact on overall survival, whereas HLA type and vaccination regimen did not. Notably, similar factors, that is, M category and localization of the primary, revealed an impact on the presence of SSTRs, whereas again HLA type and vaccination regimen did not. SSTRs were significantly more often observed in women. Notably, female patients have been reported to show better responses to anti-melanoma immunotherapies [25]; however, this subject has not yet been studied in detail. This phenomenon might be explained by the stronger immune and autoimmune reactivities observed in women compared to men, linked to the wide repertoire of immune-related genes on the X chromosome [26]. Most importantly, the present study shows a strong correlation between the rise of a specific T-cell response against survivin during vaccination and therapy outcome in terms of tumor response (*p* = 0.0008) and overall survival (*p* = 0.0077), with SSTRs being an independent predictor of patients’ survival. Interestingly, we observed an association between the presence of SSTRs and the occurrence of inflammatory reactions at the injection sites. Indeed, patients presenting these inflammatory reactions showed a trend toward a favorable survival. This observation has to be further investigated in future trials, but, nevertheless, suggests that the onset of inflammatory reactions visible at the cutaneous vaccination sites of patients treated with survivin-specific peptides might be used as an easily accessible surrogate marker for a survivin-specific T-cell response to vaccination.

Explanations are needed for the frequently reported lack of correlation between vaccine-specific T-cell responses and the clinical outcome of vaccination trials. One critical point is the immunomonitoring of vaccinated patients. To date, there is no consensus on the required assays, and standard operating procedures are missing [27]. This problem has severely limited the ability to compare the results of different vaccination trials [28]. In the present study, SSTRs were analyzed by ex vivo ELISPOT assays of peripheral blood samples of 41 vaccinated patients who consented to donate blood, revealing that 31.7 % of the analyzed patients presented a robust and reproducibly detectable survivin-specific immune response. We chose the ex vivo ELISPOT assay as the main readout due to our previous observation of (1) higher frequencies of SSTRs after in vitro stimulation, but (2) lower reproducibility, and (3) much lower correlation of the detected reactivities with the patients’ clinical course, indicating that results obtained from in vitro stimulated assays, at least in our hands, may be more difficult to interpret.

Another explanation for the lack of correlation between vaccine-specific T-cell reactivities and patients’ clinical outcome may be the choice of the target antigens [12, 14]. Tumor cell escape from immune response can be acquired by several mechanisms, with antigen loss as one of the most important ones. Unfortunately, melanocytic differentiation antigens, against which vaccination trials in melanoma have been most vigorously pursued, are ranking among this category [7]. In contrast, survivin expression is directly associated with the oncogenic phenotype of tumor cells, which ensures its maintained expression even under immuno-selective pressure [8–10, 12, 14].

We used peptides that were modified in one amino acid compared to the original epitopes in order to enhance HLA binding affinity. It has been recently suggested that vaccination with affinity-improved peptide epitopes gives rise to immune responses against the modified epitope only, but not against the wild type [29]. However, our results from epitope–MHC multimer staining in exemplary patients demonstrate that vaccination with affinity-improved survivin peptides induced T-cell responses against both, the modified as well as the native peptide.

In conclusion, the results of the present trial not only demonstrate the clinical activity of a survivin-based peptide vaccination but also show a strong correlation between the presence of anti-survivin T-cell responses and an improved clinical course of the disease as documented by progression arrest and overall survival. Moreover, survivin-specific T-cell reactivities could be shown as an independent predictor of survival in vaccinated patients. This implies that the antigen-specific T-cell reactivity (SSTR) detectable ex vivo from the patients’ blood material within the first months after onset of vaccination could be used as a surrogate marker of therapy outcome in terms of tumor response and overall survival. Thus, the attractiveness of survivin as an universal tumor antigen with oncogenic function could be translated into clinical activity in therapy-refractory, advanced melanoma patients. A survivin-specific peptide vaccination elicits an ex vivo measurable T-cell response, which renders this treatment as suitable to be applied before or together with an enhancer of T-cell response, for example, ipilimumab. Clinical trials are needed to further investigate this treatment approach.

### Electronic supplementary material

Below is the link to the electronic supplementary material.

Supplementary material 1 (PDF 145 kb)
