# Supplementary material for: Survivin-specific T-cell reactivity correlates with tumor response and patient survival: a phase-II peptide vaccination trial in metastatic melanoma
Source: Cancer Immunol Immunother. 2012 May 8;61(11):2091–103. doi: 10.1007/s00262-012-1266-9 (PMC3493663; doi:10.1007/s00262-012-1266-9)
Supplement: Supplementary file 1 — Supplementary material 1 (PDF 145 kb) [file 262_2012_1266_MOESM1_ESM.pdf]

TABLE S1: Treatment characteristics

		ITT		PP	
		61	*(100.0%)*	*55*	*(100.0%)*
**Pretreatment in stage IV**[1]	*chemotherapy*	55	(90.2%)	50	(90.9%)
	immunotherapy	24	(39.3%)	24	(43.6%)
	other	11	(18.0%)	8	(14.5%)
	1 therapy line	39	(64.0%)	35	(63.6%)
	2 therapy lines	16	(26.2%)	15	(27.3%)
	≥ 3 therapy lines	6	(9.8%)	5	(9.1%)
**Localization of primary**	*skin*	39	(64.0%)	34	(61.8%)
	mucosa	1	(1.6%)	1	(1.8%)
	uvea	16	(26.2%)	15	(27.3%)
	unknown	5	(8.2%)	5	(9.1%)
**Metastatic sites at enrolment**[1]	*skin*	28	(45.9%)	25	(45.5%)
	lymph node	41	(67.2%)	38	(69.1%)
	lung	40	(65.6%)	34	(61.8%)
	liver	35	(57.4%)	32	(58.2%)
	bone	6	(9.8%)	6	(10.9%)
	brain	9	(14.8%)	8	(14.5%)
	other visceral	15	(24.6%)	12	(21.8%)
**Number of metastatic sites at enrolment**	*1*	8	(13.2%)	8	(14.5%)
	2	19	(31.1%)	16	(29.1%)
	3	13	(21.3%)	13	(23.6%)
	≥ 4	21	(34.4%)	18	(32.8%)
**Vaccination regimen**	*regimen I*	40	(65.6%)	35	(63.6%)
	regimen II	13	(21.4%)	12	(21.8%)
	regimen III	8	(13.1%)	8	(14.5%)
**Median treatment duration** months (range)		2.9 (0.2 – 64.3+)		3.3 (0.2 – 64.3+)	
**Number of vaccinations**	*1-4*	16	(26.2%)	10	(18.2%)
	5-8	25	(41.0%)	25	(45.5%)
	9-12	12	(19.7%)	12	(21.8%)
	> 12	8	(13.1%)	8	(14.5%)

[1]Multiple entries possible. urvivin-specific T-cell reactivities were quantified by ELISPOT as described in Materials and Methods; patients showing at least one positive ELISPOT result during vaccination therapy were considered positive. [2]Best overall response was defined as the best tumor response recorded from the start of treatment until removal of the patient from the trial. [3]Survival was measured from the date of first vaccination until the date of death or disease progression, respectively; if no such event occurred, the date of the last patient contact was used as censored observation. ITT, intention to treat; PP, per protocol; CI, confidence interval; CR, complete response; PR, partial response; SD, stable disease; PD, progressive disease.

TABLE S2: Potentially treatment-related toxicities grade 3 and 4

		Patients treated	
		61	*(100.0%)*
**Grade 3 or 4 toxicity**		10	(16.4%)
Laboratory changes	hemoglobin	2	(3.3%)
	leukocytes	0	(0.0%)
	platelets	0	(0.0%)
Gastrointestine	constipation, abdominal pain	1	(1.6%)
Neurology	headache	3	(4.9%)
Cardiovascular	low blood pressure, collapse	1	(1.6%)
General/lethargy		4	(6.6%)

Toxicity was classified and graded according to CTC 2.0 (http://ctep.cancer.gov/reporting/ctc.html). Data represent the worst CTC grade experienced by each patient.

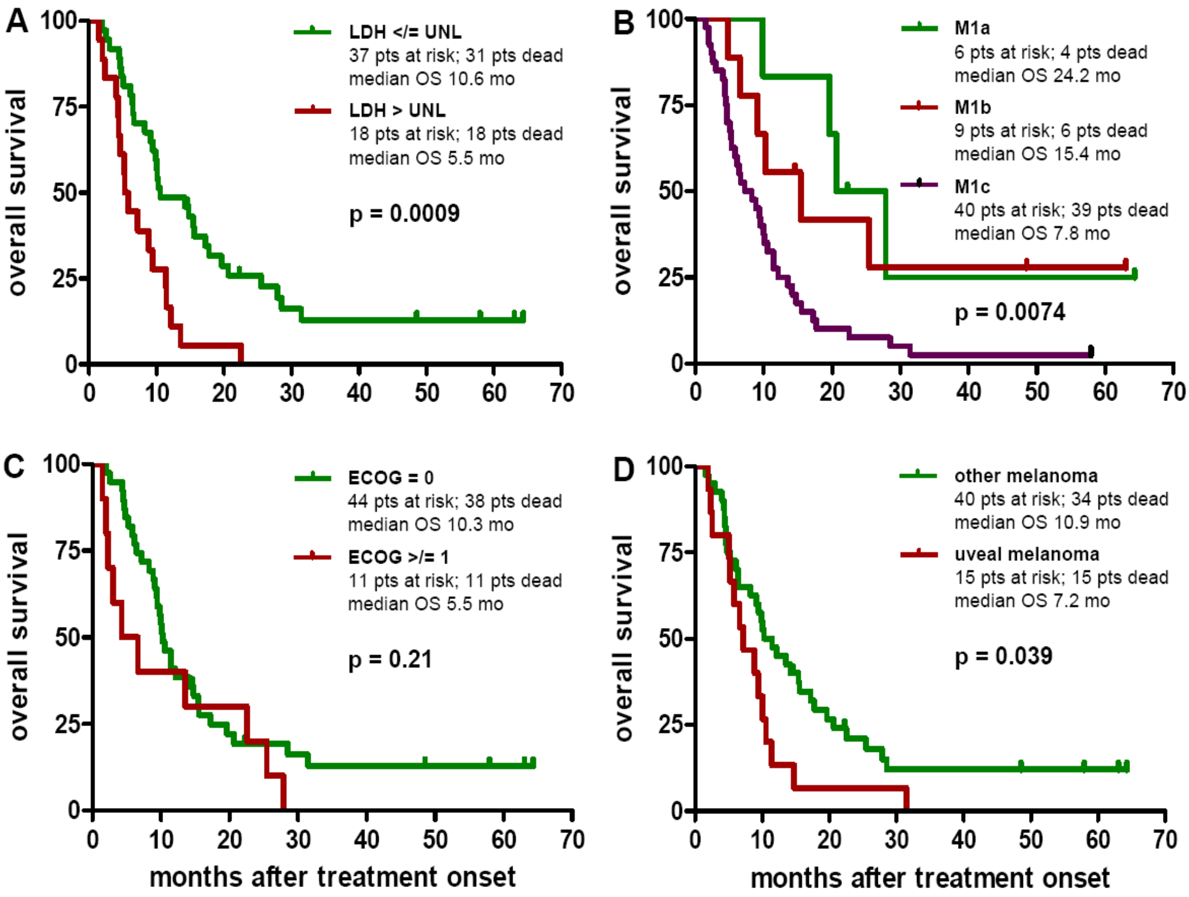

Figure S1. Kaplan Meier plots depicting the probability of overall survival (OS) of the per-protocol population (55 patients) by **(A)** serum level of lactate dehydrogenase (LDH) grouped by its upper normal limit (UNL); **(B)** M category according to AJCC criteria; **(C)** overall performance status according to ECOG classification; and **(D)** localization of the primary melanoma. Differences between groups were calculated using the log rank test; p-values are provided within the corresponding plots. Censored observations are indicated by vertical bars.
